# A game theoretic approach to balance privacy risks and familial benefits

**DOI:** 10.1038/s41598-023-33177-0

**Published:** 2023-04-28

**Authors:** Jia Guo, Ellen Wright Clayton, Murat Kantarcioglu, Yevgeniy Vorobeychik, Myrna Wooders, Zhiyu Wan, Zhijun Yin, Bradley A. Malin

**Affiliations:** 1 Department of Computer Science, Vanderbilt University, Nashville, TN 37212 USA; 2 School of Law, Vanderbilt University, Nashville, TN 37203 USA; 3 Department of Health Policy, Vanderbilt University Medical Center, Nashville, TN 37212 USA; 4 Department of Pediatrics, Vanderbilt University Medical Center, Nashville, TN 37232 USA; 5 Department of Computer Science, University of Texas at Dallas, Richardson, TX 75083 USA; 6 Department of Computer Science and Engineering, Washington University in St. Louis, St. Louis, MO 63130 USA; 7 Department of Economics, Vanderbilt University, Nashville, TN 37235 USA; 8 Department of Biomedical Informatics, Vanderbilt University Medical Center, Nashville, TN 37203 USA; 9 Department of Biostatistics, Vanderbilt University Medical Center, Nashville, TN 37203 USA

**Keywords:** Computer science, Genetic linkage study

## Abstract

As recreational genomics continues to grow in its popularity, many people are afforded the opportunity to share their genomes in exchange for various services, including third-party interpretation (TPI) tools, to understand their predisposition to health problems and, based on genome similarity, to find extended family members. At the same time, these services have increasingly been reused by law enforcement to track down potential criminals through family members who disclose their genomic information. While it has been observed that many potential users shy away from such data sharing when they learn that their privacy cannot be assured, it remains unclear how potential users’ valuations of the service will affect a population’s behavior. In this paper, we present a game theoretic framework to model interdependent privacy challenges in genomic data sharing online. Through simulations, we find that in addition to the boundary cases when (1) no player and (2) every player joins, there exist pure-strategy Nash equilibria when a relatively small portion of players choose to join the genomic database. The result is consistent under different parametric settings. We further examine the stability of Nash equilibria and illustrate that the only equilibrium that is resistant to a random dropping of players is when all players join the genomic database. Finally, we show that when players consider the impact that their data sharing may have on their relatives, the only pure strategy Nash equilibria are when either no player or every player shares their genomic data.

## Introduction

Over the past decade, direct-to-consumer genetic testing (DTC-GT) has dramatically grown in its popularity. As the amount of personal genomic data has grown, numerous companies have emerged to provide third-party interpretation (TPI) services. This is driven by the number of potential DTC-GT consumers—as of 2022, 23andme and Ancestry DNA, the two largest personal genomics companies had over 12 million and 18 million consumers, respectively^1, 2^. Of these, approximately 62% of DTC-GT consumers have sought interpretations of raw genomic data using TPI services^3^. These services support a wide variety of applications, including, but not limited to, trait analysis, personalized nutrition and diet recommendations, genealogy and ethnicity analysis, and finding relatives. Though these services are exciting, they also pose risks to consumers that can limit their uptake and adoption of such services.

For example, in 2018, the FBI arrested a suspected serial murderer known as the Golden State Killer^4^ by exploiting GEDMatch, a TPI website that, at that time, maintained a publicly available genomic database with approximately 1.5 million individuals’ DNA profiles^5^. In this case, law enforcement officers uploaded crime-scene DNA to GEDMatch and found the suspect’s third cousin, suggested by similarity in their DNA. Law enforcement officers were then able to reconstruct a family tree, trace down the suspect, and confirm the suspect’s identity by another genomic test. While this case highlighted the forensic uses of personal genomic databases, it also raised the public’s concerns over privacy with respect to this long-range familial search technique^6^. In addition, Hazel et al. pointed out that forensic investigations leveraging publicly available genomic database are unfair, underregulated, and haphazard^7^.

There has been a growing body of research responding to the case of the Golden State Killer. Edge and Coop, for instance, calculated the expected number of genetically detectable cousins one can find in the genomic database^8^. Erlich et al. further analyzed the potential for identifying an individual through their genomic data using a technique similar to that adopted by law enforcement. They showed that at the time of their investigation in 2018, approximately 60% of individuals of European descent were at risk of being identified even if they were not in the genomic database^9^, which exhibits the negative externalities incurred through online genomic data sharing. The model we introduce in this paper relies on their methods to estimate the probability of finding relatives and being re-identified.

Meanwhile, various studies have considered the extent to which current law and regulatory frameworks can address genomic privacy risks. Clayton et al. examined regulations that are applicable for genomic privacy and concluded that few, if any, are sufficient to protect genomic privacy comprehensively^10^. More specifically, the regulation of TPI services remains uncertain. For example, Guerrini et al. analyzed the potential oversight for TPI services by four US agencies and showed that the main governance of TPI services are contracts between users and TPI service providers^11^. However, when Hazel and Slobogin surveyed the privacy policies of DTC-GT companies, they discovered that most fail to comply with the Fair Information Practice Principles and the Privacy Framework proposed by the U.S. Federal Trade Commission^12^. In addition, Wan et al. appraised threats to genomic data privacy and existing sociotechnical safeguards and concluded that there is no simple solution to provide appropriate levels of genomic privacy^13^. These studies highlight the lack of protection over the genomic privacy of DTC-GT and TPI consumers.

In recognition of such challenges, numerous surveys and vignette studies have been conducted to learn more about consumers’ behaviors, motivations, and concerns with regards to the adoption of TPI services. Nelson et al. found that approximately 84% of DTC consumers used at least one TPI tool^14^. Wang et al. reported that users are highly motivated to use TPI services for ethnicity analysis and personal health implications^3^. While the majority of respondents were satisfied with the interpretation they received, 35% of the respondents were confused by the interpretation instead^14^. Besides, there are many ethical concerns on the TPI services, including inadequate informed consent, questionable clinical validity and utility, and lack of medical supervision^15^. Many concerns on DTC-GT services also apply to TPI services, including privacy, emotional toll, and general misuse of their genomic data by the company^16^. More specifically, Guerrini et al. probed public opinion on law enforcement’s access to genetic genealogy databases and found that the majority of respondents supported the access^17^. By contrast, Slobogin and Hazel also surveyed the public’s attitude, but observed that participants thought this kind of access intrusive^18^. Given the variability in users’ attitudes towards the service, as well as the existence of the privacy paradox, which describes the dichotomies between privacy attitudes and actual behavior^19, 20^, it is challenging to predict users’ behavior simply through surveys.

It should further be recognized that the aforementioned studies focus only on an individual’s perspective. However, there are circumstances under which an individual’s privacy depends not only on their own decision but also the decisions made by others. The interdependence of privacy has been studied by different research communities under different terminologies. Humbert et al. systematically summarized these research and categorized the interdependent privacy risks based on the data types^21^. According to Humbert et al., the interdependent privacy risks are either caused by the direct sharing of information involves others or the sharing of information that is correlated between individuals. The two typical sources of correlation are (1) homophily for friends on either real-life social networks or online social networks and (2) genetic inheritance. To formalize the interdependence of privacy on online social network, researchers have considered game theoretic frameworks. Biczók and Chia first proposed an Interdependent Privacy Game (IPG) model to study the adoption of third-party tools in online social networks^22^. The third-party tools often collect information that involves its users’ friends, which demonstrates the first kind of cause for the interdependent privacy risks. Subsequently, Pu and Grossklags investigated the adoption of third-party applications and generated a scale-free network to approximate the structure of real social networks^23^. Besides, Olteanu et al. investigated a more specific kind of interdependent privacy risks—the sharing of co-location information on online social network and took the time dimension into account^24^.

Humbert and colleagues first studied the interdependence of privacy in genomic data sharing^25–28^. Under the observation that genomic data is highly correlated among family members, an individual’s genomic data can be inferred through their family members. Humbert et al. quantified the genomic privacy risks^25, 27^, and developed a tool for laymen to evaluate kin genomic privacy and required no real genomic data^28^. Besides, Humbert et al. modeled the data sharing and management behaviors within a family via a game theoretic framework^26^.

It is worth noticing that the aforementioned research focus on the risks that stem from value inference attacks on genome data. As the relationship between family members becomes distant, little information about the target’s SNP values will be revealed even the SNP values of his relatives have been observed. Thus, the value inference risks significantly decrease as the degree of relatedness decreases. However, with the development of long-range familial searches, an individual can be identified by distant family members that they do not necessarily know. For example, the police have traced the Golden State Killer through 10 to 20 third-to-fourth cousins of him, most of whom he had probably never met^8^. As the number of users of TPI services increases, people start to worry about the re-identification attack enabled by the sharing decisions made by distant relatives. Besides, in reality, most TPI websites, if not all, do not provide direct access to users’ genomic data but provide genetic matching results instead. Thus, the probability of the inference attacks goes low and the re-identification attacks become the primary concern for TPI websites. Hence, in this paper, we specifically focus on the re-identification risk, which is a main difference from Humbert et al.’s model^26^.

In this paper, we introduce a game theoretic approach to characterize how potential users of TPI services may act as they weigh the tradeoff between benefits received from TPI with the privacy risks incurred in sharing one’s data. In our model, both the benefits and privacy risks of each player in this game—that is, a potential user of TPI—depend on the population of other TPI users. Different from Hembert et al.’s model^26^, we consider the data sharing behavior in the society instead of a single family. On one hand, we assume that the benefit of TPI comes from using this service to find relatives. While players in this game do not know the number of relatives they will find, they can consider the *expected* number based on genealogical information taken together with the population of other TPI users. Consequently, their benefit is an increasing function of the number of players who have chosen to use this service, which exhibits the first notable feature in our model, a network effect. On the other hand, privacy risks arise due to the possibility of the collection and use of the information in TPI by law enforcement or other third parties. Another notable feature of our model is the negative *externality* of participation decisions create for the privacy risks of others. In particular, the re-identification risks depend a great deal on a single player’s decision to join and whether one’s relatives used TPI services, as an individual can be identified by law enforcement by first identifying their relatives in TPI, as was indeed the case for the Golden State Killer and other recently solved cold cases. Nevertheless, a player can compute their privacy risks based on the number of other participants in the service as well. To the best of our knowledge, this is the first approach that captures these particular features of the decisions about whether or not to join TPI.

Through extensive simulations, we provide insights into user behavior in online genomic data sharing settings. We find that there exist three types of pure-strategy Nash equilibria in our model, which reflects what may happen in the real world: (1) no user shares genomic data with TPI (2) a small fraction of all potential users share, and (3) all users share. A Nash equilibrium is notable because it defines a solution to a game, such that no player has any incentives to change their strategy. The Nash equilibria provide us with intuitions into how the network effects and negative externalities shape players’ aggregated behaviors. We further show that our simulation results are consistent as we vary the settings of user parameters. Specifically, in our simulations, we vary users’ privacy preferences to be consistent with Westin’s distributions of privacy pragmatists, unconcerned, and fundamentalists in different years^29^ and we find no significant differences in the simulation results. We observe that the only equilibrium that is resistant to a random dropping of players is when all players share their data. Finally, we observe that social welfare (the sum of player utilities) is negative in every pure strategy Nash equilibrium except when no one shares the data (which results in zero utility). This conclusion follows from our observation that, in our model, the amount of privacy risk (that is, the probability that an individual can be re-identified) rises quickly and approaches the maximum when only a small proportion of all players share their data. This is due predominantly to the negative externality arises from long-range familial genomic inference and, consequently, is nearly independent of an individual user’s decision.

## Methods

In this section, we first introduce a game theoretic model of online genomic data sharing in a TPI service and then describe our approach to analyzing this model using simulation. Table 1 summarizes the notation used in this paper and, where relevant, the values of parameters used in our simulations. Among them, matching parameters are used when computing the probability of finding a relative, whereas model parameters are used when describing the game-theoretic model.

### A game-theoretic model of sharing genomic data with a TPI service

The TPI genomic data sharing game has a set *P* of *p* players. We assume the players are potential users of TPI services. We denote the decision by player *i* on whether to participate in the TPI service by a binary strategy $s_i \in \{0,1\}$, with $s_i = 1$ indicating the decision to share genomic data with TPI website, and $s_i = 0$ indicating the decision to not share. We use *s* to denote a profile of strategies, or *strategy profile*, of all players, while $s_{-i}$ is the collection of strategies of players other than *i*.

**Table 1:** Matching parameters and model parameters used in this paper.

Type	Notations	Definition	Value
Matching parameters	$N_0$	Number of couples from current generation	90,000,000
	*r*	Average number of children per couple	2.5
	*L*	Genome length (in Morgans)	35
	*m*	Minimum length of identity-by-descent (IBD) segments in order to be detected	0.06
	$n_s$	Number of IBD segments required to declare a match	2
	$n_u$	Minimum number of detected matches required to declare success of identification	2
	$g_{maxi}$	Relationships that can help to identify a target	5
	$g_{maxc}$	Relationships to consider when finding relatives of a target	3
Model parameters	*P*	The set of players	
	$s_i$	Player *i*’s strategy	
	$s_{-i}$	Strategies of all players except *i*	
	$b_i$	Player *i*’s valuation for finding a relative in the database	
	$v_i$	Player *i*’s net valuation for other services provided by TPI services	
	$c_i$	Player *i*’s valuation for the risk of being identified	
	*K*	Number of players who use the TPI services	
	$K_{-i}$	Number of players other than *i* use TPI services	
	*T*	A collection of types of players	
	*t*	A player type, $t \in T$	
	$n_t$	Number of types in our simulation	

There are three components in a player’s utility function. The first component is a player *i*’s *net valuation* $v_i$ for the TPI service, which is independent of others’ decisions. $v_i$ may be positive or negative, as potential users may find a service burdensome, or there may be a cost for using the service^30^.

The second component is the utility gained from finding relatives in the database. Let $b_i$ denote the value of finding a relative in the database. If we use $P(\text {relative})$ to denote the probability that a randomly selected individual in the database is a relative of the player *i*, then the expected number of relatives in the database is $K_{-i} \cdot P(\text {relative})$, where $K_{-i} = \sum _{j \in P\setminus \{i\}} s_j$ denotes the number of players other than *i* who choose to join the database. Consequently, this component of the utility function can be formally represented as $b_i s_i K_{-i} \cdot P(\text {relative}).$ We will discuss how to approximate $P(\text {relative})$ shortly.

The third component of the utility function captures the costs incurred due to privacy risk that arises when either player *i* or *i*’s relatives are in the database. We associate this privacy risk with a cost $c_i$ to player *i* and $P(\text {identified}|s_i,K_{-i})$, the probability that *i* is identified (e.g., by law enforcement) when $K_{-i}$ others have joined the database. Now, if $s_i = 1$, which means that player *i* shares genomic data, they can be identified with probability 1, and the associated cost is then $c_i$. Crucially, however, there is a probability that *i* can be identified *even if they choose not to join the database* ($s_i = 0$), but entirely as a result of the presence of their relatives in it. We use $P(\text {identified}|n_u, K_{-i})$ to denote the probability that *i* is identified if they do not join but $K_{-i}$ others do, where $n_u$ represents the number of relatives required for a successful identification. The calculation of $P(\text {identified}|n_u, K_{-i})$ will also be discussed later.

Putting everything together, the utility function of player *i* is

1 $$\begin{aligned} U_i(s_i,s_{-i}) = v_i s_i + b_i s_i K_{-i} \cdot P(\text {relative}) - c_i \left( (1-s_i) \cdot P(\text {identified}|n_u, K_{-i}) + s_i\right) .\end{aligned}$$

Since this utility function only depends on the number of players other than *i* who join TPI, $K_{-i}$, we also denote it by $U_{i}(s_i,K_{-i})$.

#### Discounting benefits

**Figure 1:**
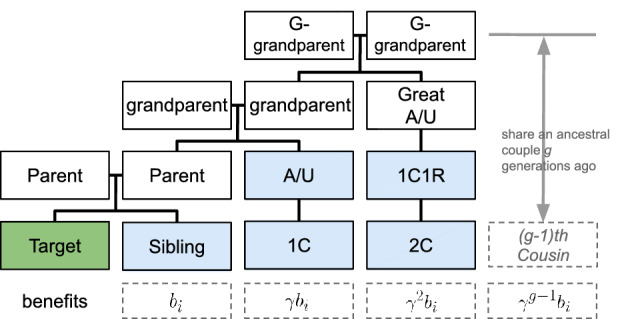
An illustration of discounting benefits for different degrees of relatedness between players. 1C: first cousin; 1C1R: first cousin once removed, and so forth. Relationships shaded in blue are considered relevant when searching for relatives.

We also consider the situation under which the benefits from finding relatives are discounting as the relatives become more distant. A discounting parameter $\gamma$ is added to each player’s valuation of the benefits. As demonstrated in Fig. 1, the benefits for a player *i* to find their $(g-1){th}$ cousins or ($(g-2){th}$) cousins once removed are $\gamma ^{g-1} b_i$. For example, the benefit from finding a sibling is $b_i$, and the benefit from finding a first cousin, aunt, or uncle is $\gamma b_i$. To sum up, if we consider a player will benefit from finding up to their second cousins, then the second component in the utility function becomes

2 $$\begin{aligned} \begin{aligned}{}&s_i K_{-i} \cdot (b_i P(\text {siblings}) + \gamma b_i P(\text {1}{st}\text { cousin}) + \gamma b_i P(\text {aunt/uncle}) + \gamma ^2 b_i P(\text {2}{nd}\text { cousin}) + \gamma ^2 b_i P(\text {1}{st}\text { cousin once removed})) \end{aligned} \end{aligned}$$

#### Types of players

In our model, we consider millions of players, which yields games that are extremely large. To make the resulting games feasible to analyze, we introduce an additional structure. Specifically, we assume that utility functions of players can be grouped into a small collection of *types* *T* and associate each player *i* with a type *t*, which is characterized by a set of parameters $(v_t,b_t,c_t)$. Consequently, the utility function of a player *i* with type *t* becomes

3 $$\begin{aligned} \begin{aligned} U_{it}(s_i,K_{-i}) = v_t s_i + b_t s_i K_{-i} \cdot P(\text {relative}) - c_t \left( (1-s_i) \cdot P(\text {identified}|n_u, K_{-i}) + s_i\right) . \end{aligned} \end{aligned}$$

#### Altruistic players

In addition to modeling players as being purely self-interested, we consider a variation with players who are altruistic. To do so, we extend the utility model to include a fourth *altruistic* component, such that a player considers the impact that their joining the TPI service has on their relatives.

We represent the set of family members for player i who are in and out of the database as $P_{in}^i$ and $P_{out}^i$, respectively. When $s_i = 1$, the utility for each player $j \in P_{in}^i$ (i.e., each family member of *i* who is in the database) increases by $b_j$ because they can find one more relative. The utility for each player $j \in P_{out}^i$, however, decreases, as this individual’s probability of being identified increases. Before player *i* joins, it requires $n_u$ relatives in the database for them to be identified. After player *i* joins, only $n_u - 1$ other relatives are required to identify them. Thus, the utility function for an altruistic player is:

4 $$\begin{aligned} U_{it}^{\text {altruistic}}(s_i,K_{-i}) = U_{it}(s_i,K_{-i}) + \beta \cdot s_i \left( \sum _{j \in P_{in}^i} b_j - \sum _{l \in P_{out}^i} c_{l} \cdot \left( P(\text {identified}|n_u-1, K_{-i}) - P(\text {identified}|n_u, K_{-i})\right) \right) , \end{aligned}$$

where $\beta \in (0, 1)$ is relative weight of the altruistic portion of this utility function.

#### Social welfare

To evaluate the efficiency of Nash equilibria, we calculate the social welfare associated with each Nash equilibrium. In our model, we define social welfare in equilibrium *s* as $W(s) = \sum _i U_i(s)$ , or the sum of utilities of all players.

### Computing the probability of finding a relative

**Figure 2:**
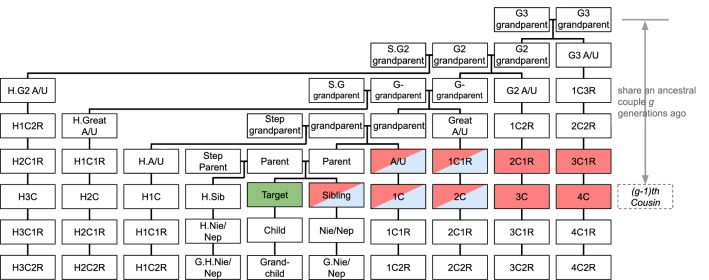
The familial relationships considered in the interdependent privacy model. We aim to uncover relatives of a targeted individual (shaded in green). 1C: first cousin; 1C1R: first cousin once removed, and so forth. Relationships shaded in blue are considered relevant when searching for relatives, while relationships shaded in red are considered when attempting to identify the target.

Our calculation of $P(\textrm{relative})$ follows the method proposed by Erlich et al.^9^. The assumptions we rely upon during the calculation are summarized as follows:

1. We do not consider the influence of half-siblings or half-cousins.
2. We assume generations are discrete and non-overlapping. For individuals in the current generation, their parents are chosen via random sampling with replacement from the previous generation.
3. There are $N_0$ couples in the current generation.
4. Each couple has *r* children ($r > 2$ in order to capture the increase in population size). At *g* generations ago, the number of couples $N_g = N_0\cdot (r/2)^{-g}$.
5. Current population contains only two generations. As shown in Fig. 2, the possible relationships between players are either cousins or once-removed cousins.
6. We do not consider the probability of finding direct ancestors, such as parents and grandparents, since in reality, most people know their direct ancestors, and usually can be identified with certainty through direct ancestors.
7. Players will not benefit from finding relatives in the database who share ancestral couples with them more than $g_{maxc}$ generations ago (relatives who are more distant than $(g_{maxc} - 1){th}$ cousins) since they are distantly related.
8. We find relatives of a target by comparing the latter’s genome with all individuals in the database and identify identity-by-descent (IBD) segments, which are segments of DNA shared by people with common ancestors. We assume *s* IBD segments are needed to declare that we have found a relative of a target. We assume an IBD segment needs to be of length $\ge m$ (in Morgan, abbreviated M) to be detected.
9. To have sufficient information to identify the target, we need to find at least $n_u$ relatives who share an ancestral couple with the target $1 \le g \le g_{maxi}$ generations ago.
10. Individuals are diploid (having two sets of chromosomes, one from each parent), and only autosomal genomes are considered (i.e., no sex chromosome is considered).

As explained in Assumption 7, $$P(\textrm{relative})$$ depends on $$g_{maxc}$$. We set $$g_{maxc} = 3$$ in our model. As shown in Fig. 2, the relationships shaded in blue are considered when computing a player’s utility of finding a relative. Since we assume there are two generations, *P*(relative$$)=$$ *P*(a player is from the current generation$$)\cdot \sum _{g=1}^{g_{maxc} } P($$the player is a $$(g-1){th}$$ cousin of the target$$)+P($$a player is from the previous generation$$)\cdot$$ $$\sum _{g=1}^{g_{maxc}-1} P($$the player is a $$(g-1){th}$$ cousin once removed of the target). As mentioned in Assumption 4, if the previous generation has a population size of $$N_1$$, then size of current generation, $$N_0 = N_1 \cdot r/2$$. Thus, *P*(a player is from the current generation) $$ =\frac{r/2 \cdot N_1}{r/2 \cdot N_1 + N_1}$$, and *P*(a player is from the previous generation$$) =\frac{N_1}{r/2 \cdot N_1 + N_1}$$.

According to Erlich et al.’s model^9^, $P \left( (g - 1){th} \text { cousin} \right)$ and $P((g - 1){th}$ cousin once removed) can be calculated given $N_0$ and several additional matching parameters, as shown in Eqs. (5) and (6). In Eq. (5), the multiplicand calculates the probability that a player *i* and any other player from the same generation in the database first shares an ancestral couple *g* generations ago. It equals the probability that the two players do not share any ancestral couple from one generation ago to $g-1$ generations ago times the probability that the two players share an ancestral couple *g* generations ago. As mentioned in Assumption 4, the number of couples at *g* generations ago is $N_g = N_0\cdot (r/2)^{-g}$. And the two players each has $2^{g-1}$ ancestral couples *g* generations ago. Assuming $2^g \ll N_g$, the probability that two players from the same generation share an ancestral couple *g* generations ago is approximately $\frac{2^{g-1}2^{g-1}}{N_g} = \frac{2^{2g-2}}{N_g}$. For every $1 \le g' \le g-1$, the probability that the two players do not share any ancestral couple $g'$ generations ago is $1-\frac{2^{2g' - 2}}{N_{g'}}$. Thus, the probability that a player *i* and any other player do not share any ancestral couple from $g-1$ generations ago to 1 generation is $\prod _{g^{'}=1}^{g-1} \left( 1- \frac{2^{2g^{'} - 2}}{N_{g'})} \right)$.

The multiplier in Eq. (5) calculates the probability that player *i* and any other player share at least $n_s$ IBD segments if they first share an ancestral couple *g* generations ago. We assume they must share at least $n_s$ IBD segments in order to be successfully detected as relatives. Thus, we need to calculate the probabilities that they share *j* IBD segments, where $0\le j \le n_s - 1$, and sum them up. If two players first share an ancestral couple *g* generations ago, they have approximately $2Lg+22$ genomic blocks to inherit independently, and the probability that each genomic block contains an IBD segment is $\frac{e^{-2mg}}{2^{2g-2}}$ (please refer to Erlich et al.’s paper for a more detailed discussion on that). We can assume the number of IBD segments follows Binomial distribution. Thus, the probability that player *i* and any other player share *j* IBD segments is $\textrm{Bin} \left( j;2Lg+22, \frac{e^{-2mg}}{2^{2g-2}} \right)$, where Bin() represents the probability mass function of a binomial distribution.

In Eq. (6), the multiplicand and multiplier are similar to Eq. (5). But as player *i* and the other player are cousins once removed and we assume player *i* is always from the later generation, we assume their shared ancestral couple is $g+1$ generations from player *i* and *g* generations from the other player. $g+1$ generations ago, the population size is $N_{g+1}$. The other player, who is from the previous generation, has $2^{g-1}$ ancestral couples *g* generations ago, and player *i* has $2^{g}$ ancestral couples $g+1$ generations ago. Thus, the probability that they share an ancestral couple $g+1$ generations from the current generation ago is $\frac{2^{2g-1}}{N_{g+1}}$.

Given the values of matching parameters and *g*, $P \left( (g - 1){th}\text { cousin} \right)$ and $P((g - 1){th}$ cousin once removed) can be considered as constants in the following computations.

5 $$\begin{aligned}{} & {} \begin{aligned} P \left( (g - 1){th}\text { cousin} \right) = \left[ \prod _{g^{'}=1}^{g-1} \left( 1- \frac{2^{2g^{'} - 2}}{N(g^{'})} \right) \frac{2^{2g - 2}}{N(g)} \right] \cdot \left[ 1- \sum _{j = 0}^{n_s-1} \textrm{Bin} \left( j;2Lg+22, \frac{e^{-2mg}}{2^{2g-2}} \right) \right] \end{aligned} \end{aligned}$$

6 $$\begin{aligned}{} & {} \begin{aligned} P \left( (g - 1){th}\text { cousin once removed} \right) = \left[ \prod _{g^{'}=1}^{g-1} \left( 1- \frac{2^{2g^{'} - 1}}{N(g^{'} + 1)} \right) \frac{2^{2g - 1}}{N(g + 1)} \right] \cdot \left[ 1- \sum _{j = 0}^{n_s-1} \textrm{Bin} \left( j;2Lg+22, \frac{e^{-2mg}}{2^{2g-2}} \right) \right] \end{aligned} \end{aligned}$$

### Computing the probability of being identified

$P(\text {identified}|n_u, K_{-i})$ denotes the probability that a player *i* can be identified through other players that use TPI services, where $K_{-i}$ is the number of other players using TPI services, and $n_u$ is the number of relatives of player *i* required to be in the database in order to successfully identify player *i*. Our calculation of $P(\text {identified}|n_u, K_{-i})$ also follows the method introduced by Erlich et al.^9^. When $n_u$ matches are required for a successful identification, the probability of being identified for player *i* equals one minus the probability that less than $n_u-1$ relatives are found from the current and the previous generation. Thus, we need to calculate the probability that *k* relatives of player *i* are found in the database, where $0 \le k \le n_u - 1$. Among the *k* relatives found, we need to calculate the probability that $k_0$ of them are $(g-1){th}$ cousins of the target, while $k - k_0$ of them are $(g-1){th}$ cousins once removed of the target, where $0\le k_0 \le k$. As we assume the target player is always from the current generation, his cousins are all from the current generation and his cousins once removed are all from the previous generation. We denote the number of players in the database from the current generation by $K_0$ and we denote the number of players in the database from the previous generation by $K_1$, then we have $K_{-i} = K_0 + K_1$. As we assume each couple has *r* children and people from the two generations have a equal probability to use TPI services, we have $K_0 = K_1 \cdot r/2$. Thus, we have $K_0 = \frac{r/2}{1+r/2} \cdot K_{-i}$ and $K_1 = \frac{1}{1+r/2} \cdot K_{-i}$. We assume players can not be identified through relatives who are more distant than $(g_{maxi} - 1){th}$ cousins. In our model, we set $g_{maxi} = 5$. As shown in Fig. 2, the relationships shaded in red are considered when computing a player’s probability of being identified. Thus, the probability that any other player from the current generation is a cousin of the target player *i* is $\sum _{g = 1}^{g_{maxi}} P((g-1){th} ~\text {cousin}))$, and the probability that any player from the previous generation is a cousin once removed of the target player *i* is $\sum _{g = 1}^{g_{maxi}-1}P((g-1){th} ~\text {cousin once removed})$. When the number of players is sufficiently large, we can assume the number of relatives of a target player *i* in the database follows Binomial distribution. Thus, the probability that the target player *i* has $k_0$ cousins in the database is $\text {Bin}(k_0; K_0, \sum _{g = 1}^{g_{maxi}} P((g-1){th} ~\text {cousin}))$. Similarly, the probability that the target player *i* has $k - k_0$ cousins once removed in the database is $\text {Bin}(k - k_0; K_1, \sum _{g = 1}^{g_{maxi}-1}P((g-1){th}~ \text {cousin once removed}) )$. To sum everything up, we have

7 $$\begin{aligned} P(\text {identified}|n_u, K_{-i}) = 1 \!- \sum _{k = 0}^{n_u-1} \sum _{k_0 = 0}^{k} \text {Bin}(k_0; K_0, \sum _{g = 1}^{g_{maxi}} P((g-1){th}~ \text {cousin})) \cdot \text {Bin}(k - k_0; K_1, \sum _{g = 1}^{g_{maxi} -1}P((g-1){th}~ \text {cousin once removed}) ) \end{aligned}$$

### Computing all pure-strategy Nash equilibria

Our analysis of the game defined above hinges on the ability to compute Nash equilibria in this game. We focus on *pure-strategy Nash equilibria (PSNE)*. Formally, a strategy profile *s* is a *pure-strategy Nash equilibrium* if for all $i \in P$, $U_i(s) \ge U_i(1-s_i,s_{-i})$ (note that in this definition we take advantage of the fact that $s_i \in \{0,1\}$). Additionally, we take advantage of the added *type* structure of our game. We say that a strategy profile *s* is a *type-symmetric Nash equilibrium* if it is a Nash equilibrium and for all types $t \in T$ and all players *i* and *j* with type *t*, $s_i = s_j$. In other words, a type-symmetric equilibrium is a Nash equilibrium in which all players with the same type play the same strategy.

To calculate PSNE in our simulations, we follow the method proposed by Daskalakis and Papadimitriou^31^ and tailor it for the binary strategy space. In this method, since the number of pure strategy profiles can be extremely large, their algorithm exhaustively searches over the partitions of strategies and examines if the partition will lead to a Nash equilibrium. A partition represents the number of players who choose each strategy. When the strategy space is binary, we can simply search among the possible number of players who choose to use TPI services.

Specifically, in our model, *p* is on the order of hundreds of millions and it is extremely time consuming to search every possible *K* between 0 and *p*. However, below (in Lemma 2) we show that it will suffice to restrict attention to TSPNE. This reduces the search space dramatically. When there are $n_t$ types of players and each type contains the same number of players, the search space reduces from $p+1$ to $n_t + 1$. The modified algorithm is shown in Algorithm 1. The correctness of Algorithm 1 is proved below.
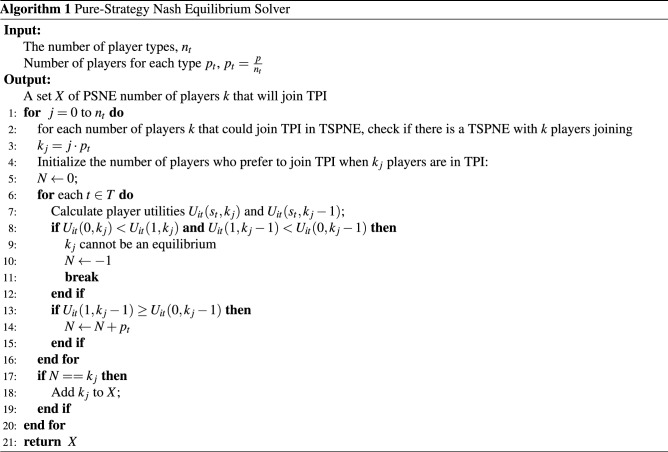


#### **Lemma 1**

*Suppose that for a player* *i*, $s_i = 1$ *is a best response to* $K_{-i} = k$, *where* *k* *is a non-negative integer. Then* $s_i = 1$ *is a* *strict* *best response if* $K_{-i} =k'$ *for any* $k' > k$.

#### *Proof*

Define $\Delta U(k) = U_i(1,k)-U_i(0,k)$. Then, $\Delta U(k) = v_i + b_i \cdot k \cdot P(\text {relative}) + c_i \cdot P(\text {identified}|k) - c_i.$ $P(\text {identified}|k)$ is increasing in *k*, and $b_i \cdot k \cdot P(\text {relative})$ is linear in *k* and, thus, strictly increasing in *k*. Consequently, $\Delta U(k)$ is strictly increasing in *k*.

Now, suppose that for some *k*, $s_i = 1$ is a best response, which means $\Delta U(k) \ge 0$. Then, since $\Delta U(k)$ is strictly increasing in *k*, for any $k' > k$, $\Delta U(k') > 0$ and, consequently, $s_i = 1$ is a *strict* best response. $\square$

#### **Lemma 2**

*Every pure-strategy Nash equilibrium* *s* *must be type-symmetric*.

#### *Proof*

We prove by contradiction. Let *k* be the number of players joining TPI in a PSNE *s*, and consider two players *i* and *j* who share the same type *t* (and, consequently, the same utility function). Let $\Delta U_{it}(k) = U_{it}(1,k)-U_{it}(0,k) \equiv \Delta U_t(k)$, since this is independent of *i*. Suppose that $s_i = 1$ and $s_j = 0$. Since *s* is a PSNE, $s_i = 1$ means that $\Delta U_t(k-1) \ge 0$. By Lemma 1, this means that $\Delta U_t(k) > 0$, which means that *j*’s best response is $s_j = 1$, a contradiction. $\square$

#### **Lemma 3**

*For any* *k*, *there is at most one pure-strategy Nash equilibrium* *s* *with* $\sum _{i} s_i = k$.

#### *Proof*

We prove by contradiction. Consider two type-symmetric PSNE *s* and $s'$ with $\sum _i s_i = \sum _i s_i' = k$ and $s \ne s'$. This means that there exist types *t* and $t'$ where $s_{it} = 1$ and $s_{jt'} = 0$, while $s_{it}' = 0$ and $s_{jt'}' = 1$. Since $s_{it} = 1$ when $k-1$ other players join, and *k* players other than *i* join in $s'$, by Lemma 1, it must be that in equilibrium, $s_{it}' = 1$, a contradiction. $\square$

#### **Theorem 1**

*Algorithm 1 returns all pure-strategy Nash equilibria.*

#### *Proof*

Algorithm 1 considers all possible values of *k* that are viable for type-symmetric pure-strategy Nash equilibria. Since by Lemma 3, each *k* is associated with at most one PSNE, it suffices to show two things for an arbitrary *k*:

1. If *k* is added to the set of Nash equilibria by the algorithm (when $N = k$), it is indeed a Nash equilibrium, and
2. If the algorithm does not add *k* to the set of Nash equilibria, there does not exist a Nash equilibrium *s* with $\sum _i s_i = k$.

The first condition is that the algorithm is *sound*, while the second condition is that it is *complete*.

We start with soundness (condition 1). Suppose that $N = k$. Since *N* is only increased if each player *i* of a particular type *t* prefers $s_i =1$ (perhaps weakly) to $s_i = 0$ when $k-1$ others join, the only concern is the possibility that some types prefer $s_i = 0$ if $k-1$ others joint, but $s_i = 1$ when *k* others join. However, this is ruled out by the condition that $U_{it}(0,k_j) < U_{it}(1,k_j)$ and $U_{it}(1,k_j-1) < U_{it}(0,k_j-1)$ (“if” statement in line 8), which explicitly concludes that in such a case *k* cannot be an equilibrium.

Next, we consider completeness (condition 2). Suppose that for a given *k*, there is a PSNE *s* with $\sum _i s_i = k$. We now show that the algorithm will necessarily add it to the set of Nash equilibria. First, note that if there is such a *s*, by Lemma 2 it must be type-symmetric and by Lemma 3 it must be unique. Thus, for each player *i* in each type *t*, it must be the case that if $s_{it} = 1$, then $U_{it}(1,k-1) \ge U_{it}(0,k-1)$, and if $s_{it} = 0$, then $U_{it}(0,k) \ge U_{it}(1,k)$. Algorithm 1 in constructing a profile $s'$ effectively assigns $s_{it}' = 1$ for any *i*, *t* with $U_{it}(1,k-1) \ge U_{it}(0,k-1)$, and assigns $s_{it}' = 0$ whenever $U_{it}(1,k-1) < U_{it}(0,k-1)$ and $U_{it}(0,k) \ge U_{it}(1,k)$ (since the alternative is ruled out by the “if” statement on line 8). Consequently, the only potential issue is that Algorithm 1 yields $N > k$, assigning $s_{it}' = 1$ to types *t* for which $s_{it} = 0$ in the actual unique PSNE *s*. Let $T'$ be the set of such types. Since *s* is a PSNE, it must be the case that for each $t \in T'$ and any individual with type *t*, both $U_{it}(1,k-1) \ge U_{it}(0,k-1)$ and $U_{it}(0,k) \ge U_{it}(1,k)$ hold, that is $s_i = 1$ is a best response when $k-1$ others join, and $s_i = 0$ is a best response when *k* others join. However, this is ruled out by Lemma 1. $\square$

### Evaluating stability of Nash equilibria

In addition to the existence and distribution of Nash equilibria, we are interested in determining which Nash equilibrium is more stable. The notion of stability we adopt here is robustness to small perturbations of player strategies. Precisely, let *s* be a TSPNE, and suppose we flip the strategies of players of $r \cdot n_t$ randomly chosen types, resulting in a new strategy profile $s'$. Next, consider best response dynamics (BRD) that begins at $s'$, defined as follows. In iteration $l=0$, let $s_l = s'$. Then in each iteration $l>0$, for each type *t* and player *i* with this type, set $s_{i,t,l}$ to be the best response to $s_{l-1}$. After a finite number of iterations, this process either reaches a fixed point $s_{\infty }$, or a cycle *C* (returning to a previously visited profile $s_l$ for some iteration *l*). Let BRD(*s*) returns either $s_{\infty }$ or *C*. We can now define stability fully formally.

#### **Definition 1**

A strategy profile *s* is *r*-stable if $s =$BRD($s'$), where $s'$ flips the player strategies in *s* of $r\cdot n_t$ randomly selected types. It is unstable otherwise.

Typically, we will simply refer to profiles as stable or unstable, with *r* specified in context.

## Results

### Simulation setup

We performed three sets of simulations. In the first set of simulations, we aim to approximate the real-world scenario and learn about whether there exists a Nash equilibrium. Furthermore, in the event that there are Nash equilibria, we aim to determine how they are distributed. In these simulations, we set *p* equal to 32.4 million, as in reality, only a small portion of people have gotten their genomic data tested and ready to share. We set the number of players in the game to be 10% of the population, which approaches the current number of DTC-GT customers. According to the most recent statistics, there are more than 26 million people who have taken at-home genomic tests by the start of 2019^32^. In addition, we set $n_t = 18{,}000$. The value of $n_t$ in these simulations is determined by the pilot experiments we conducted under each parametric setting. We examined how many Nash equilibria are captured under different values of $n_t$. If $n_t$ is too small, it can not approximate the real-world scenario well, which may miss possible Nash equilibria. However, the computing of Nash equilibria becomes more time-consuming as $n_t$ increases. When evaluating the stability of a Nash equilibrium, we set $r = 0.05$ in our simulations.

Regarding the user parameters, we set $v_i = 0$ for all types of players and $b_i$ to be uniformly distributed between 0 and 1, while the distribution of $c_i$ follows Westin’s privacy segmentation^33^. Specifically, Westin categorized consumers as “privacy fundamentalists”, “privacy pragmatists”, and “privacy unconcerned” based on their privacy preferences. The fundamentalists value privacy most while the unconcerned care little about privacy protection. The pragmatists make their decisions based on the privacy risk and the value of their information in different scenarios. Though there are a number of critiques of Westin’s segmentation, it is widely adopted when evaluating users’ privacy attitudes^34, 35^. Since surveys from different years yield different distributions of Westin’s categories^33,36^, we aim to learn if the change in privacy preferences over time affects the distribution of Nash equilibrium outcomes. We use the statistics on Westin’s categories summarized by Woodruff et al.^29^ and conduct 50 simulations for distribution of Westin’s categories in 2001, 2003 and 2014, respectively, as summarized in Table 2. As there were three surveys conducted in 2014, we calculate the average distribution of Westin’s categories for that year. We uniformly distributed $c_i$ between 0 and 20 for unconcerned, 20 and 80 for pragmatists, and 80 and 100 for fundamentalists. For example, according to statistics in 2014, 41% of players are privacy fundamentalists, then we set $0.41 \cdot n_t$ types of players to be privacy fundamentalists, and their $c_i$ values are uniformly distributed between 80 and 100. This distribution was selected based on the expectation that fundamentalists and unconcerned tend to have strong preferences and, thus, have more extreme $c_i$ values. Moreover, the $c_i$ values of privacy pragmatists should have a wider range as their preference can change among different scenarios.

**Table 2:** Distribution of Westin’s categories for various years.

Year	Fundamentalist (%)	Pragmatist	Unconcerned
2014	41	52	7
2003	26	64	10
2001	34	58	8

In the above simulations, we assume players’ valuations for finding a relative are always $b_i$. We also considered the situation under which players’ valuations for finding a relative decrease as the relatives found become more distant. In the following experiment, we examine how the value of discounting factor $\gamma$ influences the distribution of Nash Equilibria. We set $v_i = 0$ for all types of players and $b_i$ to be uniformly distributed between 0 and 1. We let the distribution of $c_i$ follow the distribution of Westin’s categories in 2014. We conduct 50 simulations respectively for $\gamma = 0.9$, $\gamma = 0.5$, and $\gamma = 0.1$, with $n_t = 18{,}000$.

In the second set of simulations, we examine the influence of user parameters on the distribution of Nash equilibria by varying the distribution of $v_i$, $b_i$, and $c_i$ respectively. For these simulations, we set *p* equal 324 million to approximate the size of U.S. population. This is because with the existence of long-range familial search, everyone in the U.S. faces the risk of being identified through their relatives, including people who do not use TPI services and even people who do not get their genomic data tested. In other words, regardless of whether people realize the risk or not, they become players in the game. Therefore, we consider the players of this game to contain the entire population in the U.S. and set the number of players to be 324 million. It is worth mentioning that the change in number of players does not affect $P(\text {relative})$ because the calculation of $P(\text {relative})$ only depends on $N_0$ and other preset matching parameters. In addition, we set $n_t = 6000$ in these simulations.

To conduct the simulation, we first assume $c_i$ is uniformly distributed between 0 and $\max (c_i)$ and vary the value of $\max (c_i)$ while keeping $b_i = 1$ and $v_i = 0$ for all types of players. We choose $\max (c_i) \in \{1, 10, 50, 100, 500, 1000\}$. Next, we set $c_i = 50$ and $v_i = 0$ for all types of players, and assume $b_i$ is uniformly distributed between 0 and $\max (b_i)$ and vary $\max (b_i)$ where $\max (b_i) \in \{0.1, 0.5, 1, 5, 10, 50\}$. Finally, we set $b_i = 0$ and $c_i = 50$ for all players, and assume $v_i$ is uniformly distributed between $\min (v_i)$ and $\min (v_i) + 100$. We choose $\min (v_i) \in \{-100, -50, -20, -10, 0\}$. We conduct 10 simulations for each value of $\max (c_i)$, $\max (b_i)$, and $\min (v_i)$.

In the third set of simulations, we adopt an uninformative parameter setting and aim to learn the distribution of Nash equilibrium when there is no prior knowledge on user preferences. More details on simulation setup and simulation results will be introduced in Appendix.

### Distribution of Nash equilibrium outcomes

**Figure 3:**
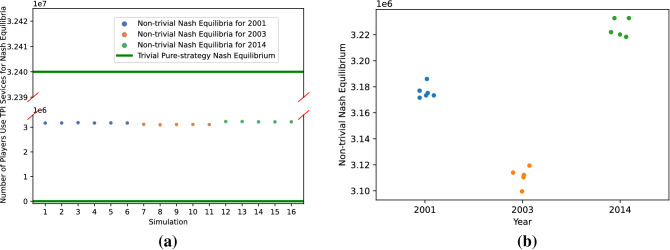
Simulation results when the parametric setting follows Westin’s privacy segmentation. (**a**) The distribution of Nash equilibria when the distribution of $c_i$ follows Westin’s categories for different years; (**b**) Interior Nash equilibria found in 50 simulations under different parametric settings.

Figure 3 depicts the Nash equilibria observed in the experiments. One noteworthy observation is that every simulation includes both the “no one joins” ($K=0$) and “all join” ($K=p$) extremes, as illustrated by the two green lines in Fig. 3a. Parametric variation appears to have no impact on the existence of the two extreme equilibria. $K=0$ is an equilibrium because in this case players there are neither network benefits (since there is no chance of finding relatives in an empty TPI service), nor negative network externalities due to privacy risks from relatives. Since $v_i = 0$ in our simulations, there is no benefit to joining the service in this case. When $K = p$, on the other hand, an individual’s decision to join the service has minimal marginal impact on their privacy risks, since it is essentially 1 for sufficiently large *p*; consequently, marginal cost of joining is negligible, whereas benefit is significant, since one can find relatives by joining TPI services.

In addition to the two extreme equilibria, we also identify a number of *interior* Nash equilibria, that is, equilibria in which the number of players joining TPI services is $0< K <p$. They are illustrated by the dots of different colors in Fig. 3a. The simulations that do not yield interior Nash Equilibria have been omitted. It is noteworthy that all interior Nash equilibria that we identify involve approximately 3 million players joining TPI, or approximately 10% of the total number of players. Figure 3b offers finer-grained observations of how interior equilibria are distributed for different parameters corresponding to the settings of Westin’s privacy segmentation for 2001, 2003, and 2014. We can see that there is not a great deal of variation in the number of players who choose to join the TPI services over time.

### Social welfare

**Figure 4:**
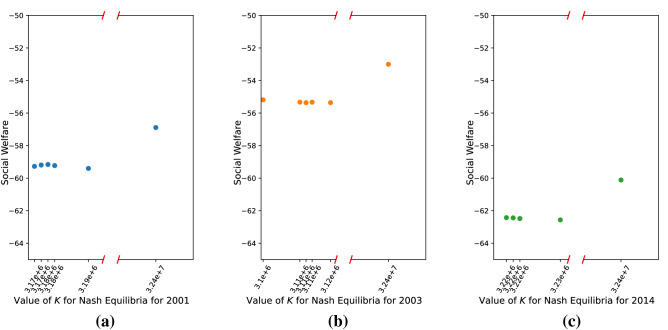
Social welfare associated with Nash equilibria found in different sets of simulations. We do not include the point when $K = 0$ in the figure, as the social welfare is always 0. *K* represents the number of players who use the TPI services. The social welfare for the extreme Nash equilibrium $K = p$ is always greater than the social welfare for interior Nash equilibria. The equilibrium in which no one joins ($K=0$), however, always has the highest social welfare.

Next we consider the social welfare of the game for the different pure strategy equilibria. Since different set of simulations are based on different parametric settings, the values of social welfare are not comparable among different set of simulations. Figure 4 presents the values of social welfare for the Nash equilibria found in different simulations. Since social welfare when $K = 0$ is always 0, we do not include this point in the figure. Surprisingly, we can observe that social welfare at the interior Nash equilibria ($0< K < p$) is dominated by social welfare of the two extreme equilibria, with $K=0$ having far higher welfare than all others. This is because the benefit term in each player’s utility functions is linear in $K_{-i}$, while the cost term increases rapidly in $K_{-i}$. The negative externality due to *others* joining approaching its maximum value with a relatively small number of joining players. Specifically, in our model, when *K* equals three million, the probability of being identified is 97.6% for a player who is not in the database. Thus, the privacy loss for this player, $c_i \cdot P(\text {identified})$ is high and close to the maximum privacy loss. On the other hand, after this point, the marginal benefits of joining rise faster than marginal cost as *K* increases, so that the extreme equilibrium at $K=p$ yields higher social utility than all interior equilibria.

### Stability

Finally, our stability analysis reveals that both of the extreme equilibria are stable. In contrast, we observe that all interior equilibria are unstable. This is somewhat surprising, and indicates that the interior equilibria are unlikely to be persistent phenomena of the system.

### Altruistic players

**Figure 5:**
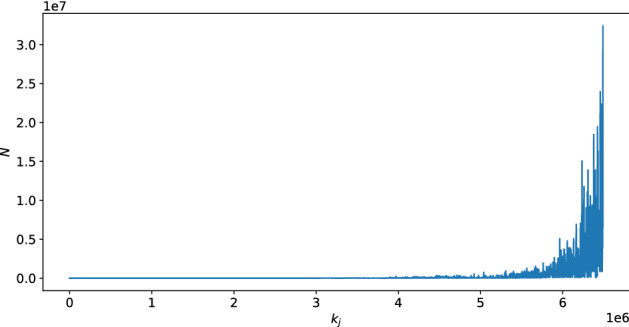
The number of players who choose to join the database (*N* in Algorithm 1) as a function of $k_j$. It can be seen that *N* fluctuates as $k_j$ increases. This occurs because players consider the utilities of their relatives.

In the variation of our model in which the players are altruistic, we find that all interior equilibria are eliminated entirely, and we only observe the two extreme equilibria. To gain intuition into this result, Fig. 5 illustrates the values of $k_j$ and *N* in Algorithm 1 from one simulation where the parametric setting follows the distribution of Westin categories in 2001. It can be seen that *N* varies with changes in $k_j$. This is because, in our simulation, we randomly select a player’s relatives. Thus, when a player considers their relatives’ utilities, their own utility fluctuates because their relatives have different values of $b_i$ and $c_i$. As a result of these fluctuations, finding $k_j$ with $N = k_j$ becomes quite unlikely.

Additionally, we observe that *N* increases quickly and reaches *p* under a small $k_j$ (when $k_j = 6,489,000$ in one simulation). Consequently, when players are altruistic, as long as there are around 6,489,000 players in the database, the best strategy for all players is to join the database.

### Discounting benefits

**Figure 6:**
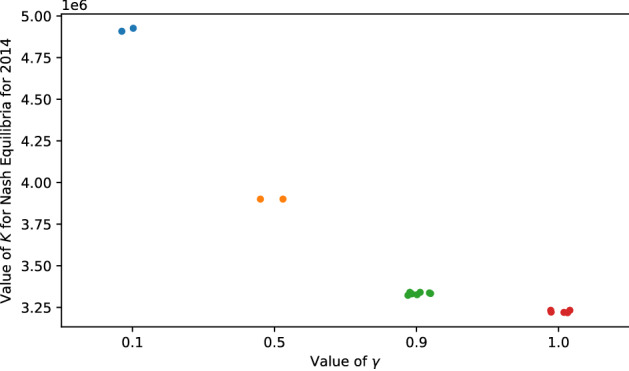
Changes in the distribution of interior Nash Equilibria as the value of $\gamma$ changes. As $\gamma$ increases, the value of *K* that leads to a Nash Equilibrium decreases.

We consider the situation in which a player’s valuation for the benefit of finding a relative discounts as the degree of relatedness decreases. While the value of $\gamma$ does not affect the existence of two extreme Nash Equilibria, $K = 0$ and $K = p$, it affects the distribution of interior Nash Equilibria. Figure 6 presents the distribution of Nash Equilibria under different values of $\gamma$. From it, we observe that as $\gamma$ decreases, the value of *K* that leads to a Nash Equilibrium increases. This is because as $\gamma$ decreases, with the same distribution of $b_i$ and $c_i$, players’ valuations for the benefits from finding relatives decrease. Thus, a Nash Equilibrium requires more players to be in the database so that the network effect balances against the privacy risks. Besides, it is noteworthy that even with $\gamma = 0.1$, the number of players who choose to use TPI services in an interior Nash Equilibrium is still small compared with the total number of players (around 15%). Thus, our main result is robust against the changes in the value of $\gamma$.

### Change in user parameters

**Figure 7:**
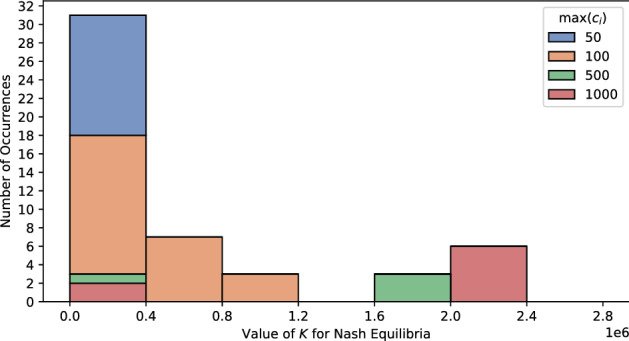
Change in Nash equilibrium with respect to $\max (c_i)$. The increase in $\max (c_i)$ pushes the value of *K* that leads to Nash equilibrium from 0 to 0.4 million to around 2 million.

Figure 7 shows the change in the distribution of interior PSNE as a function of $\max (c_i)$. It can be seen that, as $\max (c_i)$ increases, interior Nash equilibria tend to occur at higher values of *K*. We know a player will choose to join the database when $U_i(1, k) - U_i(0, k) = v_i + b_i \cdot k \cdot P(\text {relative}) + c_i \cdot P(\text {identified}|k) - c_i \ge 0$. Thus, when $c_i$ values of players are relatively lower, they tend to join the database with a small value of $K_{-i}$, and the interior Nash equilibrium is quickly reached. However, when $c_i$ values are higher, players tend to join the database with more other players in the database. Thus, the interior Nash equilibrium will happen with a higher *K* value.

For the simulations where we vary $\max (b_i)$ and $\text{min}(v_i)$, we find no interior Nash equilibria.

## Discussion

This investigation yields three main findings. First, we provide insights into the existence of Nash equilibria in the model of genomic data sharing through simulations based on a game-theoretic framework. First, we find that both extreme situations where either everyone, or no one, joins TPI, constitute Nash equilibrium outcomes in every setting we considered. In addition, we observe that interior equilibria tend to occur when the number of users of TPI services is a small fraction of the US population—specifically, around 3 million. While many factors can affect the number of users of such services, in our model, this appears to be mainly caused by the negative externality incurred. Specifically, as the number of other players who use TPI services grows, the negative externality quickly increases to a point where players suffer from the same amount of risk they would incur if they fail to use such services. In such a situation, it is clear that the best strategy for potential users is to contribute their genomic data and join TPI. Second, we observe that the optimal Nash equilibrium is realized when no one uses TPI services. Notably, when everyone is in the system uses TPI services, the social welfare is still negative. This, too, is a consequence of the negative externality of players joining the service in leaking privacy of others who have not. Third, we observe that both extreme equilibrium outcomes (everyone and no one joins) are stable, whereas interior equilibrium outcomes ($0< K < p$) are always unstable.

Our study has a number of limitations. First, for ease of computation, we assign players to a limited number of types. While this assignment significantly narrows the space needed to search for the pure-strategy Nash equilibrium, it clearly limits the diversity of preferences in the population and may thereby neglect potential Nash equilibria. We choose 18000 types in our first set of simulations, and 6000 when we vary the parametric settings. By conducting pilot studies, we are able to choose the number of types that balances the ability to capture interior Nash equilibria and the time consumption for one simulation. Second, the parameters we set for players in the game are conceptual rather than measured based on empirical observations or surveys. This is because, without performing behavioral experiments with humans, it is challenging to learn about a user’s valuations of the benefits of using TPI services and their perceived costs of privacy risks. We believe, however, that this is a crucial area for future research in the behavioral economics of privacy. Moreover, the remarkable stability of our results as we vary parameters of the utility function suggests that our overall observations are relatively robust with respect to our modeling framework. Third, we model the online genomic data sharing as a one-shot game, which lacks the ability to capture the dynamics in user interactions. In reality, users can change their decisions as their valuations for the service change. Finally, we focus specifically on the re-identification risks in our model. There are other kinds of privacy risks can be induced by the genomic data sharing on TPI websites, such as attribute inference risks. The attribute inference attack can be carried out either by insiders who have authorized access to user-shared genomic data or outsiders who obtain access through a security breach. Attackers can infer a target’s genomic data or traits by analyzing the genomic data of their relatives. As the attribute inference risks also depend on the distance between relatives and can be measured without the disclosure of actual genomic data, it can be smoothly integrated in our model and this is definitely one direction for our future work.

## Supplementary Information

Supplementary Information.

## Data Availability

Simulation results mentioned in the paper are available at https://osf.io/yjxs6/?view_only=2237f5ad4ecb4115972625a382fac91c.

## Code availibility

The code for simulations and the algorithm that solves for pure-strategy Nash equilibrium can be accessed through https://osf.io/yjxs6/?view_only=2237f5ad4ecb4115972625a382fac91c.
