# Supplementary material for: A game theoretic approach to balance privacy risks and familial benefits
Source: Sci Rep. 2023 Apr 28;13:6932. doi: 10.1038/s41598-023-33177-0 (PMC10147669; doi:10.1038/s41598-023-33177-0)
Supplement: Supplementary file 1 — Supplementary Information. [file 41598_2023_33177_MOESM1_ESM.pdf]

# Appendix

We also conduct another set of simulations when the parametric setting is uninformative. Without any assumptions on the distribution of $c_i$ in the real world, we set $v_i = 0$ for all players, $b_i$ uniformly distributed between 0 and 1, and $c_i$ uniformly distributed between 0 and 100. Similar with the second simulation we mentioned in the paper, we set $p = 324,000,000$, $n_t = 6,000$, and run 100 simulations in total. In these simulations, we want to explore the existence, distribution, and stability of Nash equilibria. Besides, we are interested in finding which Nash equilibrium leads to a social optimal. The results of the 100 simulations are presented and analyzed in the following subsections.

## Distribution of Nash Equilibrium

The distribution of pure-strategy we found in the 100 simulations is shown in Figure 1. We first observe that the two trivial Nash equilibria, $K = 0$ and $K = p$ exist in every simulation (represented by two horizontal lines).

Then, we observe that Nash equilibria may happen when only one or two types of players choose to join the database. This is because the $c_i$ values for those types of players are extremely small but the $b_i$ values are relatively large, so they will choose to join the database even there are few other players in the database.

Besides, we observe that most non-trivial Nash equilibria (53.9%) are realized when $K$ is between 0.9 million to 1.5 million so that the number of players who use the TPI services is relatively small compared to the total number of players (324 million). To investigate this issue further, we consider the utility change of a player $i$ with $v_i = 0$, $b_i = 1$, and $c_i = 100$ as the number of other players using the TPI services increases. As shown in Figure 2(a), the player's utility for joining exceeds the utility for not joining at a small $K$. This is because when players do not use TPI services, they are subject to a negative externality - the risk of being identified through the players who have used TPI services. For these players, $P(\text{identified}|k_{-i})$ increases fast as $K$ increases, and when $K = 21,885,585$, $P(\text{identified}|K_{-i})$ achieves 1, which means they endure the same amount of risk of being identified as when they use TPI services. Figure 2(b) shows the difference in utilities for players with $v_i = 0$, $b_i = 1$ and $c_i \in \{0, 1, 10, 20\}$ as $K$ increases. We can observe that for all players, $U_i^1 - U_i^0$ is monotonically increasing as $K$ increases. A player with larger $c_i$ will choose to join when there are more players who choose to use TPI services, but that $K$ is still small compared to the total number of players in the game.

We also ran simulations for the case when the players care about the utilities of their family members (i.e., the altruistic players) but found no non-trivial pure-strategy Nash equilibrium.

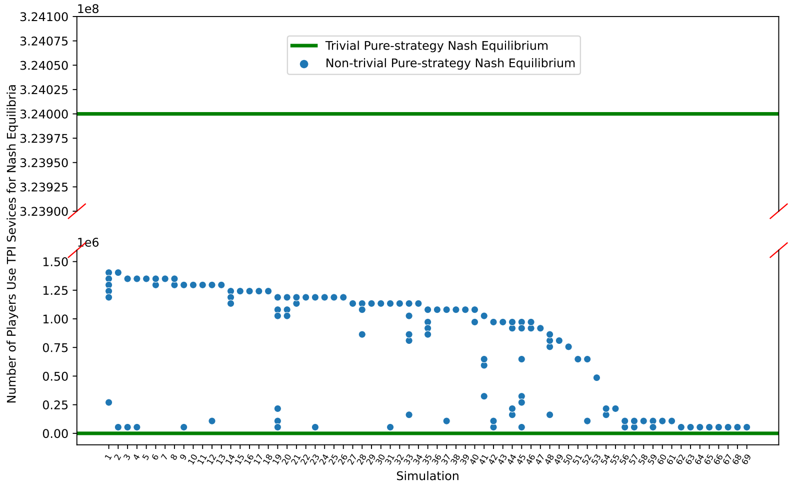


**Figure 1.** The distribution of the pure-strategy Nash equilibrium in 100 simulations. It can be seen that multiple non-trivial pure-strategy Nash equilibrium can exist in a single simulation. The simulations are ordered (in a descending manner) by the largest value of K that leads to a Nash equilibrium.

## Social Welfare

Figure 3 shows the average social welfare associated with each equilibrium in 100 simulations. It can be seen that as $K$ in each Nash equilibrium increases, the average social welfare decreases - except the case in which every player uses TPI services. To gain some intuition into why this occurs, we investigate how a player's utility changes as the number of other players who use TPI services increases. It can be seen from Figure 2(a) that the utility for the player with $c_i = 100$, $b_i = 0$, and $v_i = 0$ is the lowest when the utility for using and not using the TPI service is equivalent. When the number of other players who use the

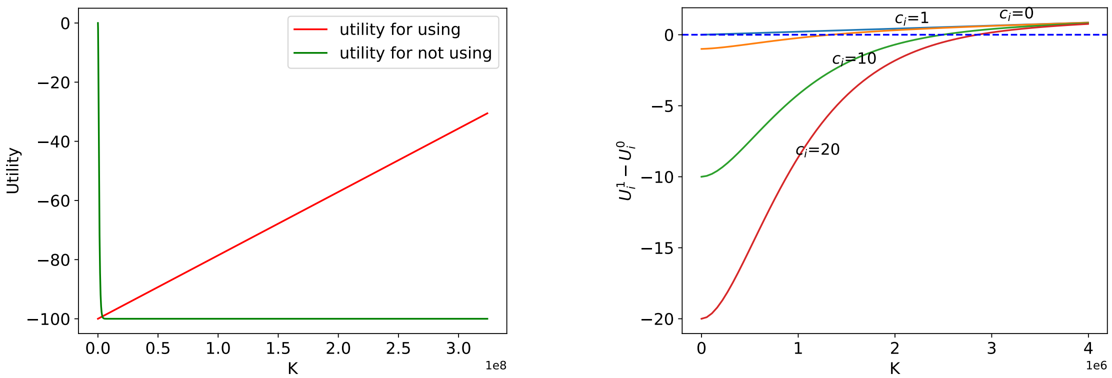


(a) Utility Change of a Player as $K$ increases.

(b) Difference in utilities as $K$ increases for players with various $c_i$.

**Figure 2.** The influence of $K$ on players' utilities. (a) For each player, the utility for using TPI services quickly exceeds the utility achieved for not using TPI services; (b) Players with higher $c_i$ values will choose to use the service when there are more other players in the database.

TPI service is lower, this player will not use the TPI service. By contrast, when the number of players who use TPI services grows, it is better for the player to use the TPI services. This is because the benefit of sharing increases and the privacy risk will be high even if they choose not to use the service.

This phenomenon is a clear demonstration of the tragedy of the commons, which frequently happens in community decision making with regards to the protection of privacy and security.

The stability for each Nash equilibrium across 100 simulations is shown in Table 1. It can be seen that the only stable Nash equilibrium is the trivial case where all players use TPI services. By contrast, the other trivial case, where no players use TPI services, is not always stable. As long as 10 out of the 6000 types of players change their minds and give TPI services a try, the game will not return to the original equilibrium. In addition, we observed that the non-trivial Nash equilibria are fragile. None of the non-trivial equilibria is resistant to both random adding or dropping. While this finding may not be sufficient to predict if the scale of TPI services will keep expanding until everyone uses them, it does indicate that the scale of the users of TPI services is unlikely to remain around 1 million.

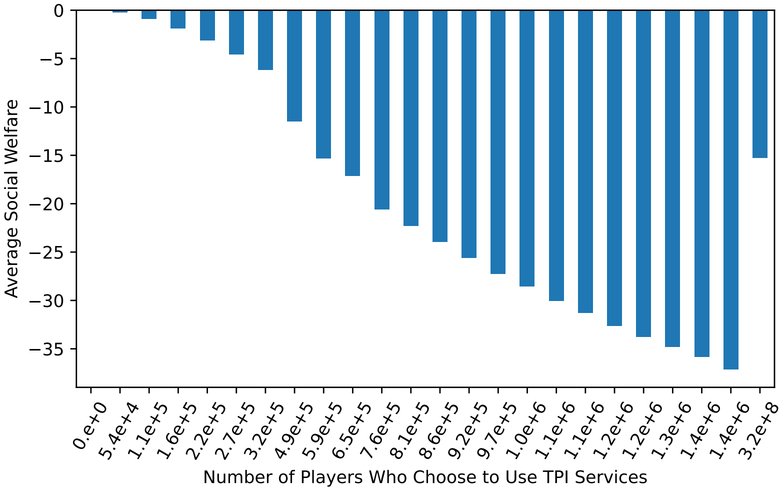


**Figure 3.** Social welfare for pure-strategy Nash equilibria. The average social welfare decreases as $K$ increases until $K = p$. As more player choose to use TPI services, the negative externalities they bring to other players keep accumulating, which leads the average social welfare to decrease. When all players choose to use TPI services, they all gain from the network effects, which increases the average social welfare.

**Table 1.** Stability of the Nash equilibrium

| *Value of k* | *Number of Occurrence* | *Resistance Added* | *Resistance Dropped* |
|---|---|---|---|
| 0 | 100 | 0.77 | NA |
| 54,000 | 20 | 0.7 | 0 |
| 108,000 | 11 | 0.91 | 0.36 |
| 162,000 | 4 | 0.5 | 0 |
| 216,000 | 4 | 1 | 0.5 |
| 270,000 | 2 | 0.5 | 1 |
| 486,000 | 1 | 1 | 0 |
| 594,000 | 1 | 0 | 0 |
| 648,000 | 4 | 0.5 | 0.25 |
| 756,000 | 2 | 0 | 0 |
| 810,000 | 3 | 0 | 0 |
| 864,000 | 4 | 0 | 0 |
| 918,000 | 5 | 0 | 0.2 |
| 972,000 | 7 | 0 | 0 |
| 1,026,000 | 4 | 0 | 0.25 |
| 1,080,000 | 9 | 0 | 0.11 |
| 1,134,000 | 10 | 0 | 0 |
| 1,188,000 | 2 | 0 | 0 |
| 1,242,000 | 6 | 0 | 0 |
| 1,296,000 | 8 | 0 | 0 |
| 1,350,000 | 7 | 0 | 0 |
| 1,404,000 | 2 | 0 | 0 |
| 324,000,000 | 100 | NA | 1 |
